# Comparison of dentoskeletal and soft tissue changes between tooth-borne and tooth-bone-borne hybrid nonsurgical rapid maxillary expansions in adults: a retrospective observational study

**DOI:** 10.1186/s12903-021-02008-x

**Published:** 2021-12-18

**Authors:** Jung-Sub An, Bo-Yeon Seo, Sug-Joon Ahn

**Affiliations:** 1 Department of Orthodontics, Seoul National University Dental Hospital, 101, Deahak-ro, Jongno-gu, Seoul, 03080 Republic of Korea; 2 Department of Orthodontics, School of Dentistry, Dental Research Institute, Seoul National University, 101, Deahak-ro, Jongno-gu, Seoul, 03080 Republic of Korea

**Keywords:** Maxillary expansion, Orthodontic anchorage, Orthodontic appliance

## Abstract

**Background:**

Despite the gradual increase in the use of rapid maxillary expansion (RME), specifically RME with the aid of skeletal anchorage in adults, there have been no reports comparing dentoskeletal and soft tissue changes between nonsurgical tooth-borne and tooth-bone-borne RMEs in adults. This study aimed to analyse differences in dentoskeletal and soft tissue changes between tooth-borne and tooth-bone-borne RMEs using a similar appliance design and the same expansion protocol in adult patients.

**Methods:**

Twenty-one patients with tooth-borne expansion (a conventional expansion screw with two premolars and two molar bands for dental anchorage [T-RME]) and the same number of patients with tooth-bone-borne hybrid expansion (a conventional expansion screw with two premolar and two molar bands for dental anchorage and four mini-implants in the palate for skeletal anchorage [H-RME]) were included. Dentoskeletal and soft tissue variables at pretreatment (T1) and after expansion (T2) were measured using posteroanterior and lateral cephalograms and frontal photographs. The sex distribution of the two groups was analysed using the chi-square test, and the change after RME in each group was evaluated using the Wilcoxon signed-rank test. Differences in pretreatment age, expansion duration, post-expansion duration, and dentoskeletal and soft tissue changes after RME between the two groups were determined using the Mann–Whitney U test.

**Results:**

There were no significant differences in the expansion protocol, pretreatment conditions, and sex distribution between the two groups. Despite similar degrees of dental expansion at the crown level between the two groups, H-RME induced increased skeletal and parallel expansion of the maxilla compared to T-RME. After expansion, H-RME demonstrated increased forward displacement of the maxilla without significant changes in the vertical dimension, while T-RME exhibited increased backward displacement of the mandible, increased vertical dimension, and decreased overbite. Both groups showed significant retroclination and extrusion of the maxillary incisors without significant intergroup differences. There were no significant soft tissue changes between the two groups.

**Conclusion:**

This study suggests that using skeletal anchorage in RME may induce increased skeletal and parallel expansion of the maxilla without significant effects on the vertical dimension.

## Background

The rapid maxillary expansion (RME) procedure, which separate the maxillary midpalatal suture orthopaedically, was popularized by Haas [1–3] and became a part of routine orthodontic treatment [4], and is still widely used to successfully correct transverse maxillary deficiency [5, 6]. In growing patients, the opening of the midpalatal suture is successfully achieved by RME with a conventional tooth-borne expander, such as the widely used Hyrax-type expander [7]. However, in adult patients, because the resistance to expansion increases as the suture matures with aging, there are concerns about the failure of the skeletal maxillary expansion using the conventional expander [4, 8]. Accordingly, a procedure such as surgically assisted RME for surgical separation of the maxilla have been proposed [9], but this present a potential risk of infection and additional costs due to an invasive operation [8].

Although several studies have reported successful nonsurgical maxillary expansion using a conventional expander in adults, the proportion of skeletal expansions out of dental expansions is lower than that in growing patients, and there are concerns about periodontal problems [8, 10–12]. Recently, with the development of skeletal anchorage, bone-borne or tooth-bone-borne hybrid expanders have been introduced, which can more directly apply orthopaedic force to the bone by fixing the expanders to the palatal bone [13, 14]. In particular, the tooth-bone-borne hybrid expander using mini-implants to reinforce the anchorage of the conventional Hyrax-type expander has received widespread attention because it obviates the need for complex surgery [8]. Successful and stable nonsurgical expansion of the maxilla can be achieved in adults using the hybrid expander [15].

Despite the recent increase in the application of RME using skeletal anchorages, studies on the differences between conventional tooth-borne expansion and expansion with the aid of skeletal anchorage in adult patients are lacking. This is because most research has been conducted on growing patients. In addition, most studies have used different expander designs with different expansion protocols, which make it difficult to compare research results. Furthermore, soft tissue changes resulting from RME have not been investigated in adults. Knowledge of the difference in treatment effects between tooth-borne and tooth-bone-borne RMEs in adults, while using a similar appliance design and expansion protocol, may provide clinicians with valuable information on how to expand the maxilla effectively and efficiently in adult patients. The purpose of this retrospective study was to analyse differences in dentoskeletal and soft tissue changes in tooth-borne and tooth-bone-borne RMEs using a similar appliance design and the same expansion protocol in adult patients.

## Methods

### Patients

To analyse differences in dentoskeletal and soft tissue changes in tooth-borne and tooth-bone-borne RMEs using a similar appliance design and the same expansion protocol, we included adult patients aged 18 years or older who were diagnosed with a transverse maxillary deficiency at the Department of Orthodontics, Seoul National University Dental Hospital from 2009 to 2020 and who underwent RME in this study. Patients who underwent maxillary expansion using a Hyrax-type tooth-borne conventional expander or tooth-bone-borne hybrid expander and who had a complete series of posteroanterior (PA) and lateral cephalograms and frontal photographs at pretreatment (T1) and after expansion (T2) were included. The pretreatment images were used for diagnostic purposes, and the images at T2 acquired at least 6 weeks after cessation of expansion before commencing the second phase of treatment were used for re-evaluation. Cephalograms and frontal photographs were acquired in the resting lip and natural head positions, and all cephalograms were acquired under the same conditions using an Asahi CX-90SP II system (Asahi Roentgen, Kyoto, Japan). Patients with the following conditions were excluded from this study: (1) craniofacial syndrome; (2) a history of trauma; (3) temporomandibular disorders; (4) a history of orthodontic treatment; and (5) gingival recession or possible bony dehiscence around the anchor teeth (maxillary first premolars and first molars). The study protocol was approved by the institutional review board of the university (IRB no. S-D20190027).

Based on the abovementioned criteria, 42 patients were included in this study. Twenty-one patients who underwent RME using a Hyrax-type conventional expander were categorised into the tooth-borne RME (T-RME) group, and 21 patients who underwent maxillary expansion using expanders to obtain anchors from the teeth and mini-implants were classified into the tooth-bone-borne hybrid RME (H-RME) group. Based on a previous study [16], a power analysis was performed using G*Power 3.1 (Heinrich-Heine, Düsseldorf, Germany) [17], and at least 20 patients per group were needed to determine the difference between the two groups in terms of skeletal expansion of the maxilla with an alpha error of 0.05 and a power of 0.8.

### Expansion protocol

**Fig. 1:**
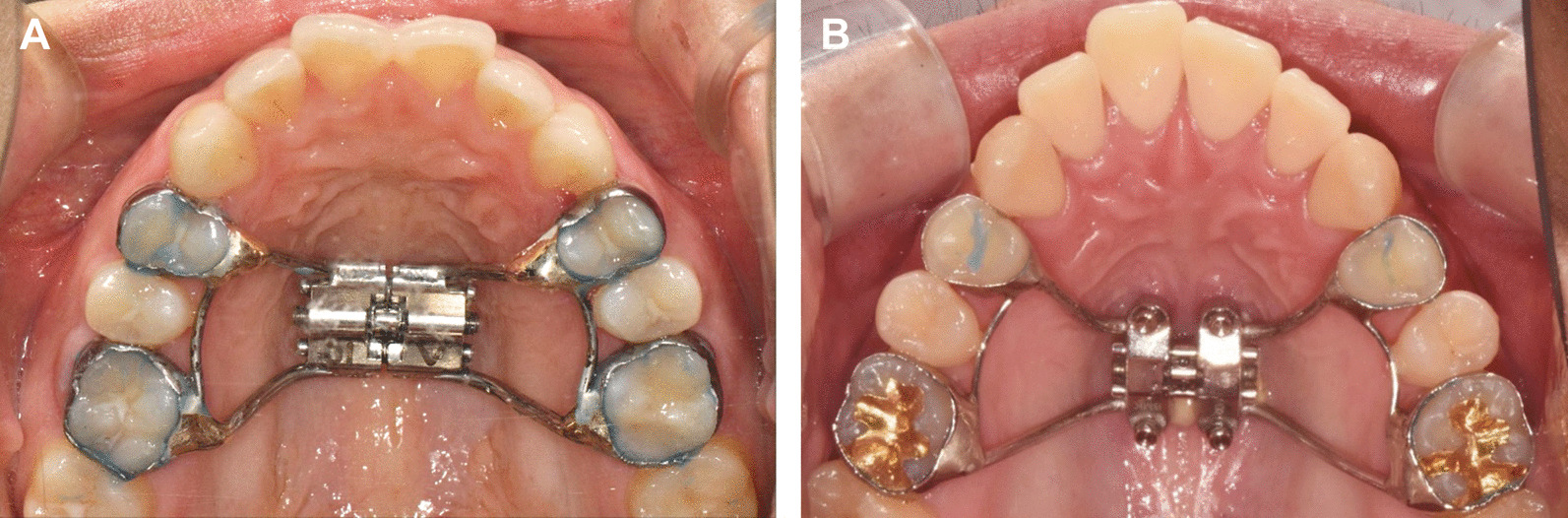
Design of expanders used in this study. **A** Tooth-borne expander with an expansion screw connected with two premolar and two molar bands for dental anchorage. **B** Tooth-bone-borne hybrid expander with an expansion screw connected with two premolar and two molar bands for dental anchorage and four mini-implants in the palate for skeletal anchorage

For all patients, an expander was fabricated in the same laboratory in the usual way after fitting the bands to the maxillary first premolars and first molars. A conventional expansion screw (Hyrax, Dentaurum GmbH & Co. KG, Ispringen, Germany) was used for the expander of the T-RME group, and an expansion screw with four holes for mini-implants (MSE I, Biomaterials Korea, Inc., Seoul, Korea) was used for the expander of the H-RME group (Fig. 1). Patients in the T-RME group started activation immediately after expander cementation, whereas in the H-RME group, four mini-implants with a diameter of 1.5 mm and a length of 11 mm (OAS-T1511, Biomaterials Korea, Inc., Seoul, Korea) were placed with the patients under local anaesthesia after expander cementation, and expansion was started two weeks after the delivery of the expander.

Both groups were instructed to activate two quarter turns per day (0.4 mm widening per day) at the start of expander activation and to reduce to one quarter turn per day (0.2 mm widening per day) after it was presumed that the midpalatal suture was opened. During the expansion period, patients were recalled at weekly intervals to evaluate expansion progress, and the opening of the midpalatal suture was primarily estimated through the appearance of midline diastema, as previously described [4]. In both the groups, the transverse maxillary deficiency was overcorrected by activating the expander until the buccal inclination of the maxillary molar palatal cusp contacted with the lingual inclination of the buccal cusp of the mandibular molar—just before the scissors bite relationship was formed. When it was judged that sufficient expansion was obtained, the expansion screw was fixed, and the expander was maintained for more than six weeks. After expansion (T2), the skeletal opening of the midpalatal suture was verified using the PA cephalogram.

### Measurements

**Fig. 2:**
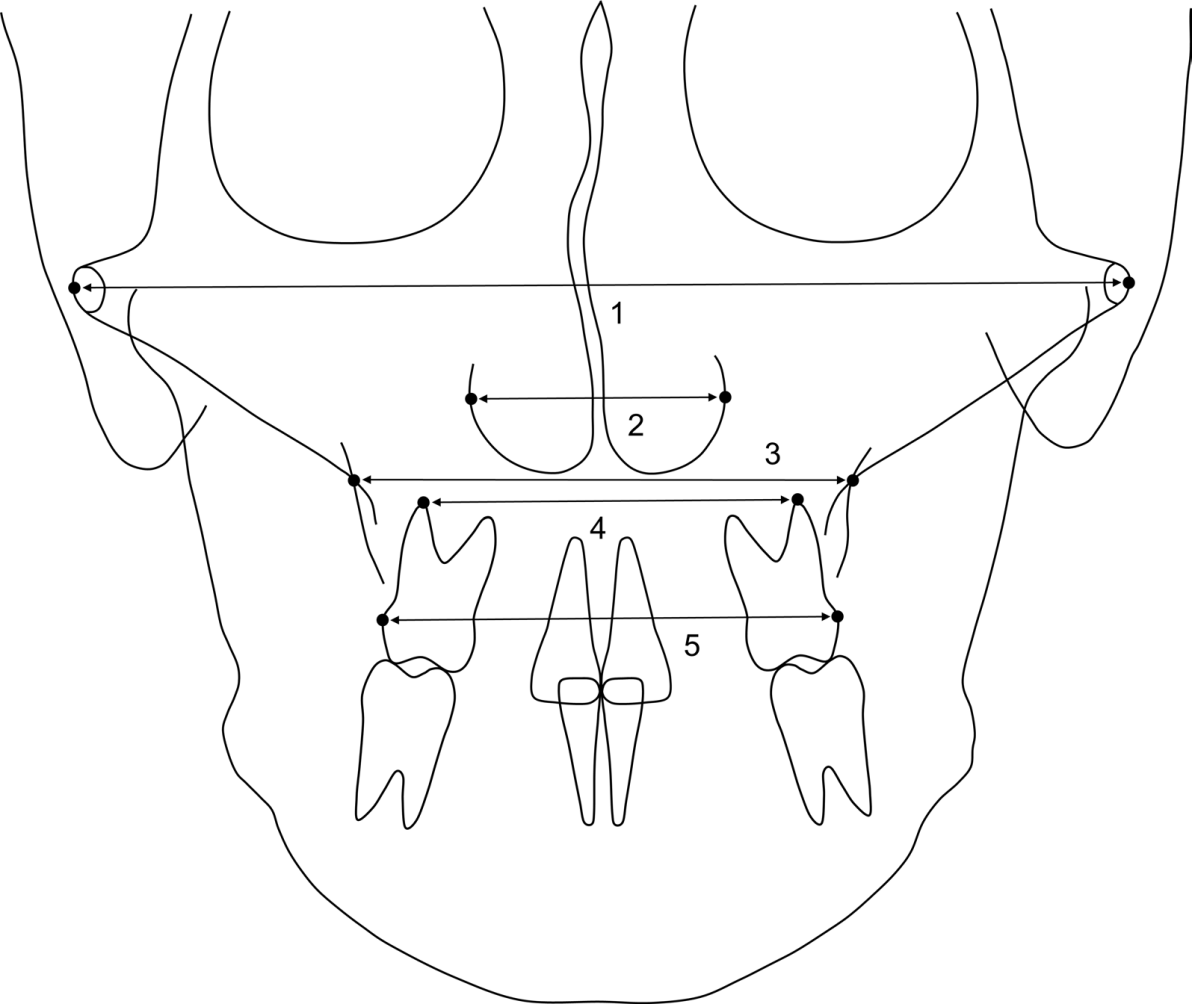
Transverse dentoskeletal variables assessed in the posteroanterior cephalogram. (1) facial width (mm): the distance between the left and right zygia (the most lateral aspect of the zygomatic arch); (2) nasal width (mm): the longest distance between left and right lateral bony walls of the nasal cavity; (3) maxillary width (mm): the distance between left and right jugal points (intersection of the maxillary tuberosity and outline of the zygomatic buttress); (4) intermolar root width (mm): the distance between left and right buccal root tips of the maxillary first molars; (5) intermolar crown width (mm): the distance between the most lateral points on the buccal surfaces of the maxillary first molar crowns

**Fig. 3:**
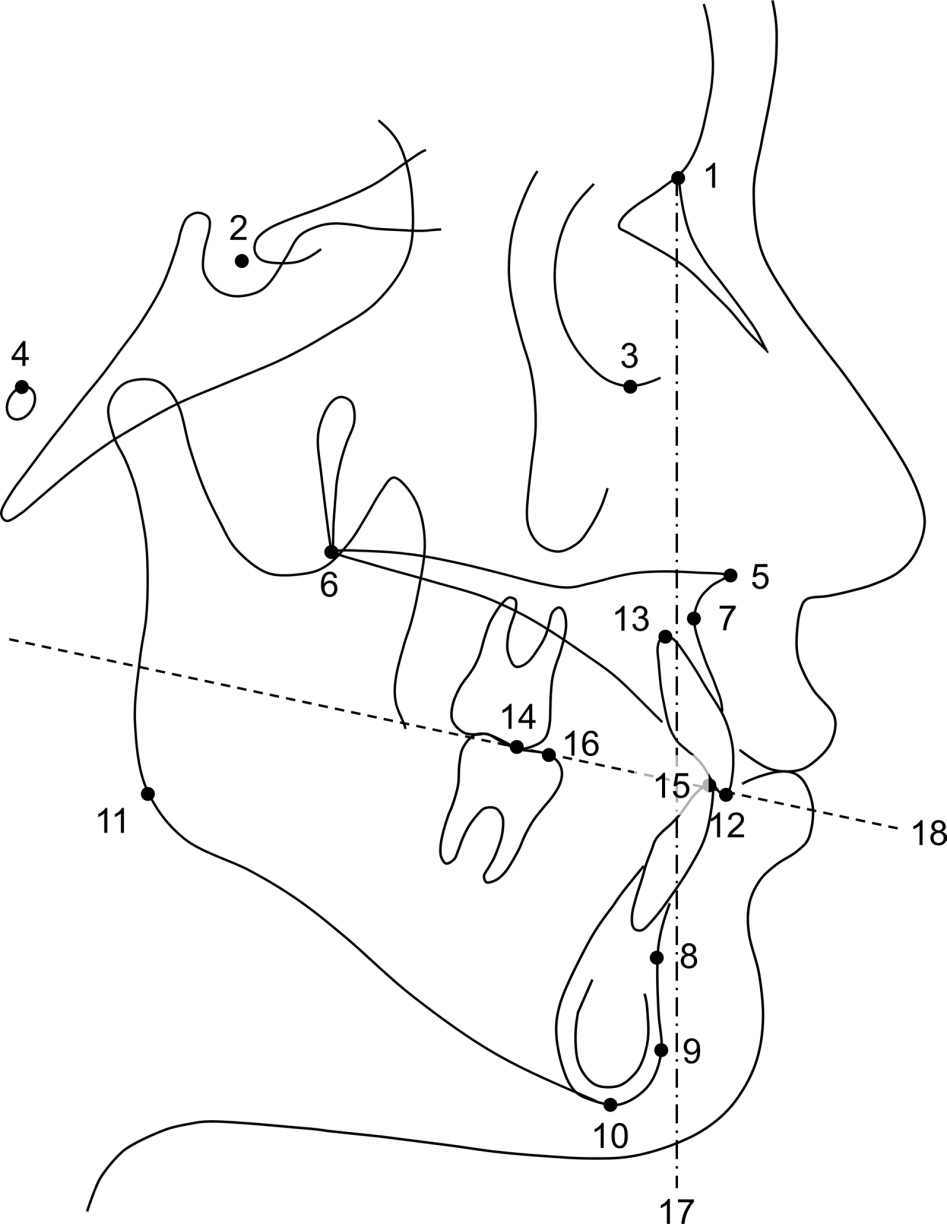
Landmarks and the reference planes assessed in the lateral cephalogram. (1) nasion; (2) sella; (3) orbitale; (4) porion; (5) anterior nasal spine; (6) posterior nasal spine; (7) A point; (8) B point; (9) pogonion; (10) menton; (11) gonion; (12) maxillary incisal edge; (13) maxillary incisor apex; (14) maxillary first molar mesiobuccal cusp; (15) mandibular incisal edge; (16) mandibular first molar mesiobuccal cusp; (17) nasion perpendicular plane: a line perpendicular to the Frankfort horizontal plane and passing through the nasion; (18) occlusal plane: a line connecting the midpoint of the incisal edges of the maxillary and mandibular incisors to the midpoint of the mesiobuccal cusps of the maxillary and mandibular first molars

**Fig. 4:**
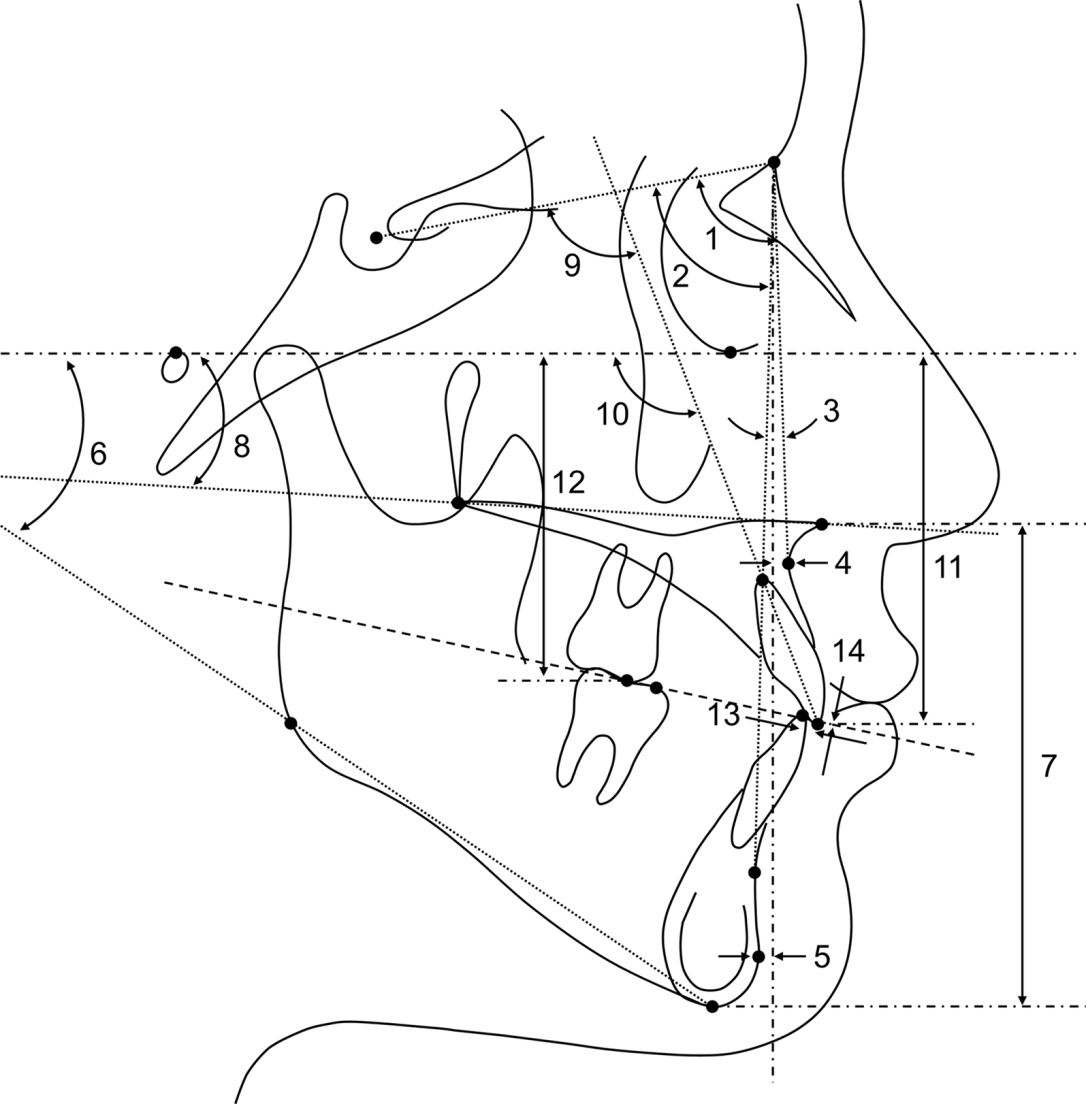
Anteroposterior and vertical dentoskeletal variables assessed in the lateral cephalogram. (1) sella–nasion-A point (SNA, °); (2) sella–nasion-B point (SNB, °); (3) A point-nasion-B point (ANB, °); (4) A point to nasion perpendicular (A to N perp, mm); (5) pogonion to nasion perpendicular (Pog to N perp, mm); (6) Frankfort-mandibular plane angle (FMA, °); (7) lower anterior facial height (LAFH, mm): the distance between the anterior nasal spine and menton parallel to the nasion perpendicular; (8) palatal plane angle (°); (9) maxillary incisor to sella–nasion plane angle (U1 to SN, °); (10) maxillary incisor to Frankfort horizontal plane angle (U1 to FH, °); (11) maxillary incisal edge to Frankfort horizontal plane (U1 IE to FH, mm); (12) maxillary first molar mesiobuccal cusp to Frankfort horizontal plane (U6 MBC to FH, mm); (13) overjet (mm); (14) overbite (mm)

**Fig. 5:**
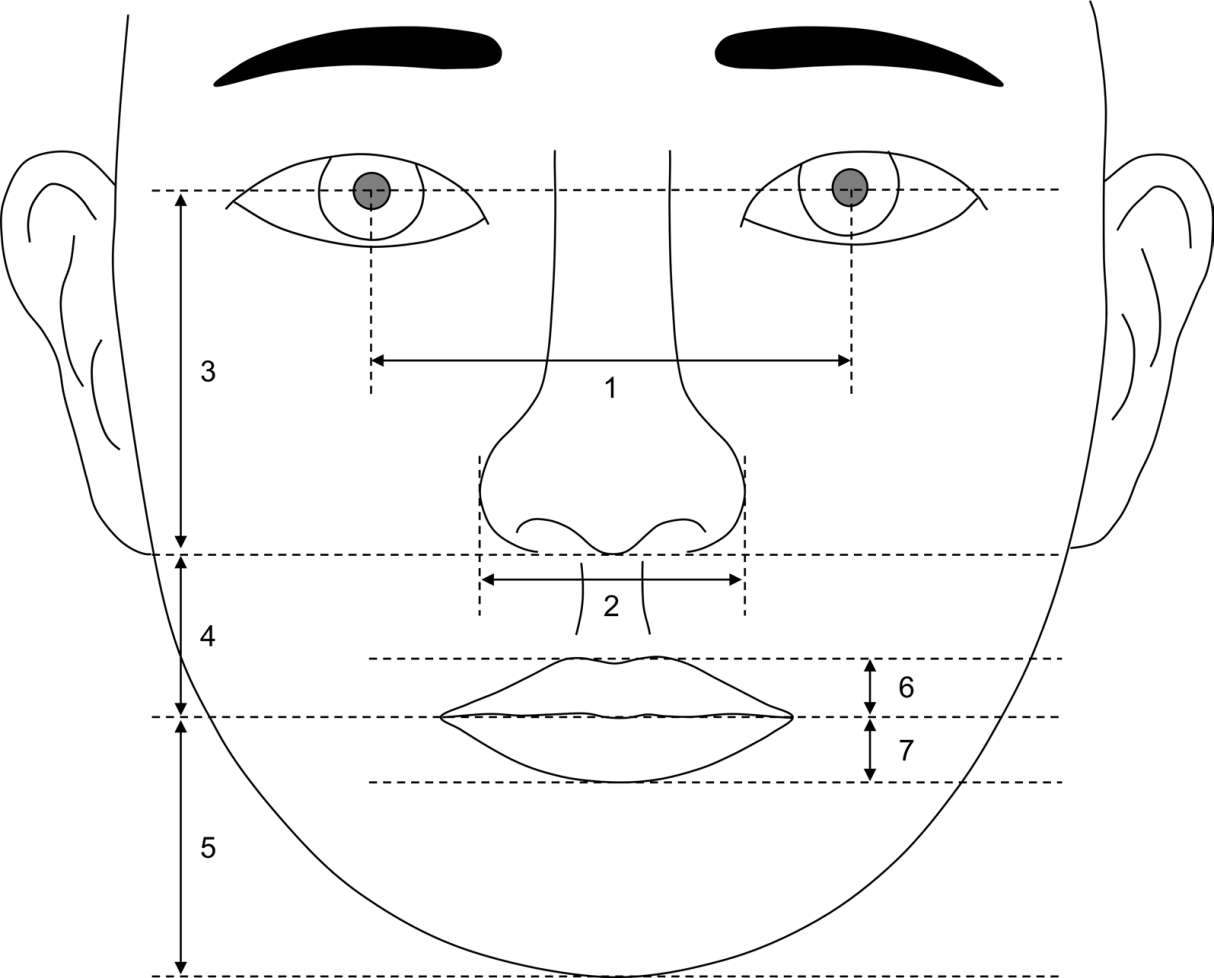
Soft tissue variables assessed in the frontal photograph. (1) interpupillary distance: the distance between left and right pupils; (2) alar width: distance between left and right alars. (3) nose length: distance between the midpoint of the pupils and subnasale; (4) upper lip length: distance between subnasale and stomion; (5) lip chin length: distance between stomion and menton; (6) upper lip vermilion: labrale superius to stomion; (7) lower lip vermilion: stomion to labrale inferius. Vertical measurements including nose length, upper lip length, lip chin length, upper lip vermilion, and lower lip vermilion were measured as a distance parallel to the vertical bisector of the pupils. The unit is the percentage of the interpupillary distance

Figures 2, 3, 4 and 5 show the dentoskeletal and soft tissue variables used in the present study. Dentoskeletal variables were measured using V-Ceph 8.0 (Osstem Implant, Seoul, Korea) on PA and lateral cephalograms, and soft tissue variables [18] were measured using a picture archiving and communication system viewer (Infinitt Healthcare, Seoul, Korea) on frontal photographs. Considering the size difference between the photographs, the soft tissue variables are presented as a percentage of the interpupillary distance. For each group, the amount of change from RME was calculated by subtracting the values of the variables at T1 from those at T2. All measurements were performed by one investigator who was blinded to the patient information, and the same investigator re-measured the records of nine randomly selected patients to evaluate intra-examiner reliability. The intraclass correlation coefficients of all measurements exceeded 0.954.

### Statistical analysis

The difference in sex distribution between the two groups was analysed using the chi-square test. The demographic (pretreatment age, expansion duration, and post-expansion duration), dentoskeletal, and soft tissue variables were tested using the Shapiro–Wilk test; however, the variables did not satisfy the normality. The significance of changes in dentoskeletal and soft tissue variables by RME in each group were analysed using the Wilcoxon signed-rank test. The Mann–Whitney U test was used to analyse the differences in pretreatment age, expansion duration, post-expansion duration, and dentoskeletal and soft tissue changes between the two groups. All analyses were performed using IBM SPSS Statistics 25 (IBM Corp., Armonk, NY, USA), and the significance level was set at α = 0.05.

## Results

Patient demographic information is presented in Table 1. No significant differences in sex distribution, pretreatment age, expansion duration, and post-expansion duration were found between the two groups.

**Table 1:** Demographics of the patients included in this study

Demographics	T-RME	H-RME	Significance
Subjects (% of total)	21 (50.0)	21 (50.0)	
Sex (% of group)			
Male	8 (38.1)	3 (14.3)	0.159^†^
Female	13 (61.9)	18 (85.7)	
Pretreatment age (years)	21.54 ± 2.59	21.97 ± 6.49	0.178^‡^
Expansion duration (days)	26.57 ± 13.37	30.95 ± 13.09	0.178^‡^
Post-expansion duration (months)	2.91 ± 0.59	2.74 ± 0.91	0.186^‡^

Data are either presented as number (%) or mean ± standard deviation

T-RME, the group who received rapid maxillary expansion (RME) procedure with the conventional tooth-born expander; H-RME, the group underwent RME using the tooth-bone-borne hybrid expander

^†^The chi-square test was used to analyse the significance of differences between the two groups

^‡^The Mann–Whitney U test was used to analyse the significance of differences between the two groups

In this study, both appliances expanded intermolar crown width by about 5.5 mm, and the increase in intermolar crown width was not significantly different between the two groups (Table 2). After RME, both expanders significantly increased the transverse dentoskeletal variables with the exception of facial width, but the expansion pattern was significantly different between the two groups (Table 2). Facial width was not significantly increased in either group, but the increase in nasal width, maxillary width, and intermolar root width was greater in the H-RME group than in the T-RME group.

**Table 2:** Changes in the transverse dentoskeletal variables after rapid maxillary expansion (RME)

Dentoskeletal variables	T-RME				H-RME				Intergroup *p *value^‡^
	T1	T2	Change	Intragroup *p *value^†^	T1	T2	Change	Intragroup *p *value^†^	
Facial width (mm)	140.78 ± 5.03	140.83 ± 5.50	0.04 ± 1.26	0.366	141.46 ± 6.22	141.65 ± 6.29	0.19 ± 0.63	0.211	0.772
Nasal width (mm)	34.23 ± 3.16	34.90 ± 3.32	0.67 ± 1.19	0.023	33.34 ± 2.25	35.64 ± 2.46	2.30 ± 1.11	< 0.001	< 0.001
Maxillary width (mm)	66.52 ± 3.58	67.88 ± 3.77	1.35 ± 1.41	< 0.001	67.52 ± 3.69	70.31 ± 3.94	2.79 ± 1.59	< 0.001	0.003
Intermolar root width (mm)	50.62 ± 2.68	53.43 ± 3.14	2.82 ± 2.09	< 0.001	51.11 ± 3.55	55.18 ± 4.19	4.07 ± 1.92	< 0.001	0.024
Intermolar crown width (mm)	59.18 ± 2.81	64.97 ± 3.37	5.79 ± 1.89	< 0.001	59.05 ± 4.06	64.37 ± 5.13	5.32 ± 2.05	< 0.001	0.222

Data are presented as mean ± standard deviation

T-RME, the group who received RME procedure with the conventional tooth-born expander; H-RME, the group underwent RME using the tooth-bone-borne hybrid expander; T1, pretreatment; T2, after RME (at least 6 weeks after fixation of the expander); Change, change in each variable after RME

^†^The Wilcoxon signed-rank test was used to analyse the significance of changes in the variables within a group

^‡^The Mann–Whitney U test was used to analyse the significance of differences in changes between the groups

Table 3 shows changes in anteroposterior and vertical dentoskeletal variables after RME. After expansion, the mandible was positioned more posteriorly relative to the maxilla in both groups (A point-nasion-B point [ANB]), and there was no significant difference in the maxillo-mandibular relationship changes between the two groups (ANB). However, the two expanders had different effects on the positions of the maxilla and mandible. After expansion, H-RME induced significant forward displacement of the maxilla (sella–nasion-A point [SNA] and A to nasion perpendicular [A to N perp]) without a significant effect on the mandible, but T-RME induced significant backward displacement of the mandible (sella–nasion-B point [SNB] and pogonion to nasion perpendicular [Pog to N perp]) without a significant effect on the maxilla.

**Table 3:** Changes in the anteroposterior and vertical dentoskeletal variables after rapid maxillary expansion (RME)

Dentoskeletal variables	T-RME				H-RME				Intergroup *p *value^‡^
	T1	T2	Change	Intragroup *p *value^†^	T1	T2	Change	Intragroup *p *value^†^	
*Skeletal variables*									
SNA (°)	78.80 ± 3.66	79.44 ± 3.22	0.64 ± 1.39	0.073	79.92 ± 3.04	80.44 ± 3.25	0.52 ± 0.98	0.021	0.960
SNB (°)	77.51 ± 3.58	76.86 ± 3.73	− 0.65 ± 0.97	0.014	78.07 ± 4.94	77.56 ± 4.69	− 0.51 ± 1.28	0.060	0.890
ANB (°)	1.29 ± 2.11	2.58 ± 2.22	1.29 ± 1.42	< 0.001	1.85 ± 3.48	2.88 ± 3.10	1.04 ± 0.99	0.001	0.950
A to N perp (mm)	− 1.90 ± 2.95	− 1.37 ± 2.63	0.53 ± 1.37	0.052	− 1.43 ± 3.70	− 0.09 ± 3.98	1.34 ± 1.39	0.001	0.005
Pog to N perp (mm)	− 6.30 ± 6.66	− 8.43 ± 7.13	− 2.13 ± 1.76	< 0.001	− 6.51 ± 10.26	− 6.33 ± 10.38	0.17 ± 2.33	0.821	0.002
FMA (°)	29.96 ± 4.28	31.24 ± 4.49	1.28 ± 1.03	< 0.001	32.01 ± 6.96	31.91 ± 7.16	− 0.10 ± 1.29	0.715	0.001
LAFH (mm)	76.92 ± 7.12	78.56 ± 6.91	1.64 ± 1.18	< 0.001	78.51 ± 5.21	78.29 ± 5.23	− 0.22 ± 2.18	0.889	< 0.001
Palatal plane angle (°)	1.33 ± 2.98	1.25 ± 2.77	− 0.08 ± 1.17	0.821	1.37 ± 4.12	0.79 ± 3.93	− 0.58 ± 1.27	0.063	0.170
*Dentoalveolar variables*									
U1 to SN (°)	103.19 ± 6.43	100.45 ± 6.51	− 2.74 ± 2.37	< 0.001	107.25 ± 7.59	103.21 ± 6.93	− 4.03 ± 3.41	< 0.001	0.163
U1 to FH (°)	112.76 ± 6.22	109.88 ± 6.47	− 2.88 ± 2.07	< 0.001	116.15 ± 7.88	112.70 ± 7.24	− 3.46 ± 3.35	0.001	0.505
U1 IE to FH (mm)	61.42 ± 3.57	62.50 ± 3.73	1.08 ± 0.85	< 0.001	62.10 ± 3.79	62.59 ± 3.77	0.49 ± 2.07	0.035	0.333
U6 MBC to FH (mm)	54.85 ± 3.51	55.43 ± 3.76	0.58 ± 1.16	0.046	55.79 ± 2.85	55.74 ± 3.22	− 0.05 ± 1.74	0.664	0.314
Overjet (mm)	2.76 ± 2.72	2.94 ± 2.52	0.18 ± 1.13	0.715	3.27 ± 3.78	3.66 ± 3.32	0.39 ± 1.24	0.085	0.285
Overbite (mm)	0.44 ± 2.74	− 0.14 ± 2.88	− 0.58 ± 1.06	0.033	0.02 ± 3.95	0.14 ± 3.67	0.13 ± 1.19	0.211	0.019

Data are presented as mean ± standard deviation

T-RME, the group who received RME procedure with the conventional tooth-born expander; H-RME, the group underwent RME using the tooth-bone-borne hybrid expander; T1, pretreatment; T2, after RME (at least 6 weeks after fixation of the expander); Change, change in each variable after RME; SNA, sella–nasion-A point; SNB, sella–nasion-B point; ANB, A point-nasion-B point; A to N perp, A point to nasion perpendicular; Pog to N perp, pogonion to nasion perpendicular; FMA, Frankfort-mandibular plane angle; LAFH, lower anterior facial height; U1 to SN, maxillary incisor to sella–nasion plane angle; U1 to FH, maxillary incisor to Frankfort horizontal plane angle; U1 IE to FH, maxillary incisal edge to Frankfort horizontal plane; U6 MBC to FH, maxillary first molar mesiobuccal cusp to Frankfort horizontal plane

^†^The Wilcoxon signed-rank test was used to analyse the significance of changes in the variables within a group

^‡^The Mann–Whitney U test was used to analyse the significance of differences in changes between the groups

After expansion, the vertical dimension was significantly increased only in the T-RME group (Frankfort-mandibular plane angle [FMA]) and lower anterior facial height [LAFH]), but was maintained in the H-RME group, resulting in a significant intergroup difference (FMA and LAFH). Both expanders did not significantly change the inclination of the palatal plane (palatal plane angle) following expansion.

In both groups, the maxillary incisors were significantly retroclined (maxillary incisor to sella–nasion plane angle [U1 to SN] and maxillary incisor to Frankfort horizontal plane angle [U1 to FH]) and extruded (maxillary incisal edge to Frankfort horizontal plane [U1 IE to FH]) after expansion without intergroup differences. Although the maxillary first molars were significantly extruded only in the T-RME group, there was no significant difference between the two groups (maxillary first molar mesiobuccal cusp to Frankfort horizontal plane [U6 MBC to FH]). While there was no significant change in overjet following maxillary expansion, overbite decreased only in the T-RME group, inducing a significant intergroup difference in overbite.

Table 4 shows the differences in soft tissue changes between the two groups after RME. Both expanders significantly increased the alar width without an intergroup difference. After expansion, the nose length and the upper lip length were significantly increased in the H-RME group and T-RME group, respectively, but there was no significant difference between the two groups for each variable.

**Table 4:** Changes in the soft tissue variables after rapid maxillary expansion (RME)

Soft tissue variables	T-RME				H-RME				Intergroup *p *value^‡^
	T1	T2	Change	Intragroup *p *value^†^	T1	T2	Change	Intragroup *p *value^†^	
Alar width (%)	58.91 ± 3.93	59.79 ± 3.75	0.88 ± 1.73	0.023	60.79 ± 3.74	61.98 ± 4.06	1.18 ± 1.52	0.004	0.624
Nose length (%)	79.77 ± 5.22	79.74 ± 5.66	− 0.02 ± 2.07	0.848	81.41 ± 4.91	82.39 ± 4.97	0.98 ± 2.32	0.025	0.204
Upper lip length (%)	36.67 ± 3.44	37.50 ± 3.17	0.84 ± 1.78	0.030	37.15 ± 5.03	37.53 ± 4.59	0.38 ± 1.75	0.434	0.505
Lip chin length (%)	73.88 ± 5.09	74.55 ± 5.68	0.67 ± 3.04	0.394	78.07 ± 9.26	77.13 ± 8.76	− 0.94 ± 3.65	0.375	0.232
Upper lip vermilion (%)	14.98 ± 1.93	15.09 ± 2.15	0.11 ± 1.02	0.476	15.53 ± 1.88	15.18 ± 1.83	− 0.35 ± 1.02	0.181	0.213
Lower lip vermilion (%)	18.33 ± 3.04	18.37 ± 2.64	0.04 ± 1.53	0.958	19.21 ± 2.98	19.60 ± 2.91	0.39 ± 1.34	0.192	0.538

Data are presented as a percentage of the interpupillary distance in the form of mean ± standard deviation

T-RME, the group who received RME procedure with the conventional tooth-born expander; H-RME, the group underwent RME using the tooth-bone-borne hybrid expander; T1, pretreatment; T2 after RME (at least 6 weeks after fixation of the expander); Change, change in each variable after RME

^†^The Wilcoxon signed-rank test was used to analyse the significance of changes in the variables within a group

^‡^The Mann–Whitney U test was used to analyse the significance of differences in changes between the groups

## Discussion

Although several studies have reported successful maxillary expansion in adult patients through nonsurgical expansion using a conventional expander [10–12, 19], there have been concerns about potential side effects, such as buccal tipping and root resorption of anchor teeth, periodontal problems, low success rate, and low stability [8, 10, 20]. Our study showed that in both T-RME and H-RME groups, the maxillary arch was successfully expanded, and the opening of the midpalatal suture was verified through the PA cephalogram at T2, as well as the presence of midline diastema after expansion. In addition, no periodontal side effects such as gingival recession of anchor teeth were observed in either group, which may be because periodontally compromised patients were not included in either group.

This study demonstrated no significant difference in sex distribution, expansion duration, observation duration, and amount of dental expansion between the two groups (Tables 1, 2), indicating that the pretreatment conditions and treatment processes were not significantly different between the two groups.

Although the amount of dental expansion was similar, H-RME induced more expansion in the nasal and maxillary areas than T-RME (Table 2). In addition, the increase in the intermolar root width was significantly larger in the H-RME group than in the T-RME group, indicating that the two maxillary halves expanded more in parallel in the H-RME group than in the T-RME group (Table 2). These results are similar to those of previous studies on adolescent patients [16, 21–23]. Considering the similar expander design and the same expansion protocol, the differences in dentoskeletal changes may be due to anchorage reinforcement using mini-implants in the H-RME group.

Skeletal expansion may not be effective in the upper part of the face above the nasal area in adult patients, because both expanders did not significantly influence facial width (Table 2). This differs from the findings of a study on adolescent patients in which the tooth-bone-borne hybrid expander provided greater expansion than the tooth-borne expander, but showed significant orbital level expansion with both expanders [23]. This difference is presumed to be due to the increased resistance to expansion in adults, as the circummaxillary sutures gradually mature with aging [8].

The two expanders influenced the anteroposterior maxillary position differently after RME. H-RME induced significant forward displacement of the maxilla after expansion, whereas T-RME did not significantly influence the maxillary position (Table 2). Forward displacement of the maxilla by disruption of the circummaxillary sutures after RME has been reported [24–26]. More skeletal expansion by H-RME may have a stronger effect on the maxilla and surrounding sutures, resulting in a secondary forward displacement of the maxilla. On the other hand, less skeletal effects of T-RME on the circummaxillary sutures may not significantly influence the anteroposterior position of the maxilla.

H-RME did not significantly change the anteroposterior mandibular position and vertical dimension, but T-RME induced significant backward displacement of the mandible and increased the vertical dimension after expansion (Table 3). These changes may be associated with differences in the expansion patterns between the two expanders. Less parallel expansion of maxillary molars by T-RME may lead to premature contact between the maxillary and mandibular molars, resulting in clockwise rotation of the mandible, which in turn, may induce an increase in the vertical dimension and backward displacement of the mandible after expansion.

Interestingly, there was no significant difference in ANB angle change after expansion between the two expanders despite different expansion effects on the maxillary and mandibular positions (Table 3). This is because more forward displacement of the maxilla by H-RME and more backward displacement of the mandible by T-RME may have a similar effect on the overall anteroposterior maxillo-mandibular relationship after expansion.

Both expanders significantly retroclined and extruded maxillary incisors after expansion, but there was no significant difference between them (Table 3). This may be associated with the backward and downward movement of the maxillary incisors that occurs during spontaneous closure of the midline diastema caused by RME. Because there was no significant difference in the amount of dental expansion between the two groups, similar movements in the maxillary incisors may be expected between them. However, both expanders changed the vertical position of the maxillary molars after expansion. T-RME significantly extruded the maxillary molars after expansion, but H-RME did not significantly change the vertical position of the maxillary molars (Table 3). Less parallel dental expansion by T-RME may partly explain the extrusion of the maxillary molars, but more parallel dental expansion by H-RME may help to maintain the vertical position of the maxillary molars.

Despite the significant retroclination of the maxillary incisors, there was no significant change in the overjet after expansion between the two expanders. This is because changes in the maxillo-mandibular relationship (anterior displacement of the maxilla by H-RME and posterior displacement of the mandible by T-RME) may offset overjet changes due to retroclination of the maxillary incisors (Table 3). Overbite was significantly decreased after expansion only in the T-RME group, which may be associated with a significant increase in vertical dimension by T-RME compared to that by H-RME (Table 3).

Table 4 shows changes in soft tissue variables after RME. Both expanders significantly increased alar width after expansion, while H-RME and T-RME significantly increased nose length and upper lip length, respectively. However, all the changes were too small to be statistically significant between the two groups (Table 4). Considering that the interpupillary distance of adults is about 60 mm [27], such a change of about 1% results in a soft tissue change of less than 1 mm. Therefore, soft tissue changes after RME may not be clinically relevant.

The results of the present study demonstrated that H-RME led to more skeletal and parallel expansion of the maxilla than T-RME in adult patients; therefore, the use of a tooth-bone-borne hybrid expander may be more appropriate when a more definite skeletal expansion is required in adult patients with a transverse maxillary deficiency. In the case of the conventional tooth-borne expander in adult patients, more dental expansion with less skeletal expansion may appear when compared with the hybrid expander. However, since clinically successful maxillary arch expansion can be expected, the conventional Hyrax expander may be used as an alternative for adult patients who cannot undergo invasive mini-implant insertion. In addition, both expanders influenced the vertical skeletal dimensions differently. H-RME did not significantly influence the vertical dimension, but T-RME significantly increased the vertical dimension after the RME procedure; therefore, the hybrid expander is more suitable for adult patients whose vertical skeletal change is undesirable.

This study showed that the anteroposterior jawbone relationships may be influenced differently by the two expanders. Although the magnitude was small, a significant anterior displacement of the maxilla by H-RME (forward displacement at the A point by approximately 1.3 mm) and backward displacement of the mandible (backward displacement in pogonion by approximately 2.1 mm) by T-RME were observed. Therefore, it should be noted that RME procedures may influence the original skeletal anteropsoterior maxillo-mandibular relationships in adult patients with transverse maxillary deficiency.

This retrospective study has limitations in that two-dimensional images, such as cephalograms and photographs, were used. Such a two-dimensional image has a problem in that image distortion and volume change cannot be evaluated. Therefore, a prospective study using three-dimensional or digital images such as cone-beam computed tomoragphy or stereophotogrammetry is considered necessary in the future [28, 29].

## Conclusions

The results of this study suggest that the tooth-bone-borne hybrid expander may be more suitable for adult patients who need more skeletal expansion without significant effects on the vertical dimension, whereas the conventional tooth-borne expander may be used as an alternative for adult patients who cannot undergo invasive procedures. Further studies using three-dimensional or digital images are needed to clarify these results.

## Data Availability

The datasets used and/or analysed during the current study are available from the corresponding author upon reasonable request.

## Abbreviations

RME: Rapid maxillary expansion
PA: Posteroanterior
T-RME: Tooth-borne rapid maxillary expansion
H-RME: Tooth-bone-borne hybrid rapid maxillary expansion
SNA: Sella–nasion-A point
SNB: Sella–nasion-B point
ANB: A point-nasion-B point
A–N perp: A point to nasion perpendicular
Pog–N perp: Pogonion to nasion perpendicular
FMA: Frankfort-mandibular plane angle
LAFH: Lower anterior facial height
U1–SN: Maxillary incisor to sella–nasion plane angle
U1–FH: Maxillary incisor to Frankfort horizontal plane angle
U1 IE–FH: Maxillary incisal edge to Frankfort horizontal plane
U6 MBC–FH: Maxillary first molar mesiobuccal cusp to Frankfort horizontal plane
